# Synthesis and Spectroscopic Characterization of Two Tetrasubstituted Cationic Porphyrin Derivatives

**DOI:** 10.3390/molecules16075807

**Published:** 2011-07-08

**Authors:** Antonio E.H. Machado, Weverson R. Gomes, Diesley M.S. Araújo, Hércules S. Miglio, Leonardo T. Ueno, Rodrigo De Paula, José A.S. Cavaleiro, Newton M. Barbosa Neto

**Affiliations:** 1 Laboratório de Fotoquímica, Instituto de Química, Universidade Federal de Uberlândia, P.O. Box 593, CEP 38400-902 Uberlândia, Minas Gerais, Brazil; 2 Centro de Ciências Agrárias, Universidade Federal do Espírito Santo, Alegre, Espírito Santo, Brazil; Email: herculesmiglio@hotmail.com; 3 Faculdade de Ciências Integradas do Pontal, Universidade Federal de Uberlândia, Ituiutaba, Minas Gerais, Brazil; Email: tsuyoshi@pontal.ufu.br; 4 Centro de Formação de Professores, Universidade Federal do Recôncavo da Bahia, Amargosa, Bahia, Brazil; Email: rodrigodepaula@ufrb.edu.br; 5 Departamento de Química, Universidade de Aveiro, Aveiro, Portugal; Email: jcavaleiro@ua.pt; 6 Instituto de Física, Universidade Federal de Uberlândia, P.O. Box 593; CEP 38400-902 Uberlândia, Minas Gerais, Brazil; Email: newtonfisico@gmail.com

**Keywords:** cationic porphyrin derivatives, MAOS, UV-Vis spectroscopy, fluorescence spectroscopy, DFT and TD-DFT, electronic states, solvent effects

## Abstract

An imidazolium tetrasubstituted cationic porphyrin derivative (the free base and its Zn(II) complex) with five-membered heterocyclic groups in the *meso*-positions were synthesized using microwave irradiation, and the compounds obtained characterized by ^1^H-NMR and mass spectrometry. We observed that under microwave irradiation the yield is similar to when the synthesis is performed under conventional heating, however, the time required to prepare the porphyrins decreases enormously. In order to investigate the electronic state of these compounds, we employed UV-Vis and fluorescence spectroscopy combined with quantum chemical calculations. The results reveal the presence, in both compounds, of a large number of electronic states involving the association between the Soret and a blue-shifted band. The Soret band in both compounds also shows a considerable solvent dependence. As for emission, these compounds present low quantum yield at room temperature and no solvent influence on the fluorescence spectra was observed.

## 1. Introduction

Porphyrins form a class of molecules with key roles in many important biological processes and have also proven to be versatile in numerous applications, such as photodynamic therapy [1], catalysis [2], photonics [3], energy conversion [4], chemical sensors [5] and many others. In particular, cationic porphyrins have several interesting features which make them attractive photosensitizers in a variety of medical applications [6,7,8]. Their systematic study has as a general goal the correlation of their electronic and structural characteristics with a specific physical chemical property. In fact, porphyrins are largely studied mainly because they permit relatively easy structural manipulation in a great variety of forms, e.g., modification of the central ion, axial and *meso* substitutions, *etc.* This is advantageous when the aim is to tune physical properties to yield an appropriate response for a specific application. Consequently, the synthesis and characterization of new types of porphyrins has become a very rich and necessary research field in many technological branches like material sciences, medicine, engineering and others.

Particularly, imidazolyl and imidazolium porphyrins have received great attention, after Milgrom´s work on the synthesis of tetraimidazolylporphyrin (TIP), which shows proton conducting properties [9]. Despite the efforts, the synthesis afforded low yields and no further studies were performed. To solve this problem, *N*-substituted imidazolylporphyrin derivatives were prepared in good yields, but accompanied by atropoisomers, which necessitates a difficult and tedious work up [10]. Even so, most of reports involving imidazolylporphyrin derivatives are related to supramolecular chemistry [11,12,13]. For instance, five-membered *meso*-substituted porphyrins are normally employed as NLO materials due to their asymmetry [14], and, in several cases, the structure must contain the pair of donor/acceptor moieties to give the desired NLO response.

**Figure 1:**
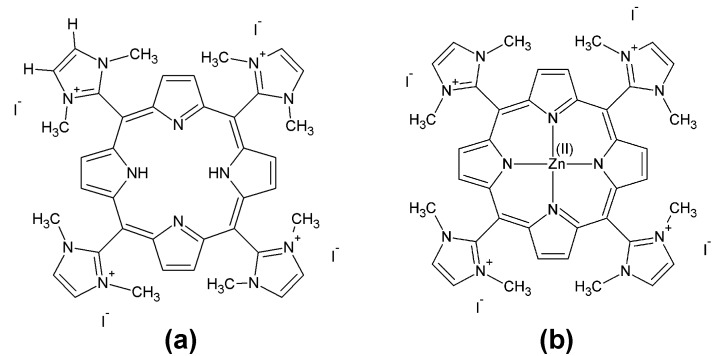
Representation of the molecular structures: (**a**) [H_2_-TDMImP]I_4_; (**b**) [Zn(TDMImP)]I_4_.

Therefore, one of our purposes in this work was to prepare materials through microwave irradiation with the aim of achieving fast syntheses and easy work ups. Also, the methylation step is useful because cationic porphyrins have received great attention due to their features. Moreover, once methylated, the materials attain high symmetry avoiding the development of atropoisomers. The compounds under study are the free base 5,10,15,20-tetrakis-(1,3-dimethylimidazolium-2-yl) porphyrin tetraiodide salt, [H_2_-TDMImP]I_4_, and its corresponding zinc (II) complex, [Zn(TDMImP)]I_4_ (Figure 1). It is important to emphasize that we could, for the first time, ascertain the catalytic efficiency of the manganese (III) complex of free base cationic derivative, obtaining promising results when it is compared with the common phenyl-substituted porphyrin derivatives [15].

Moreover, this work presents results related to spectroscopic characterization combined with quantum chemical calculations for the above mentioned free base and its Zn(II) complex. In order to partially fulfill this task, a systematic investigation with a broad amount of techniques and approaches was performed. We employed a set of experimental techniques such as Proton Nuclear Magnetic Resonance (^1^H-NMR), mass spectrometry, absorption and emission spectroscopy. The use of these techniques aims at providing information on porphyrin structure in solution, band gap characteristics, role of solvent polarity and emission quantum yield. Quantum chemical calculations were performed to support the experimental data, mainly those related to excitation spectra.

## 2. Results and Discussion

### 2.1. Synthesis and Characterization

**Scheme 1:**
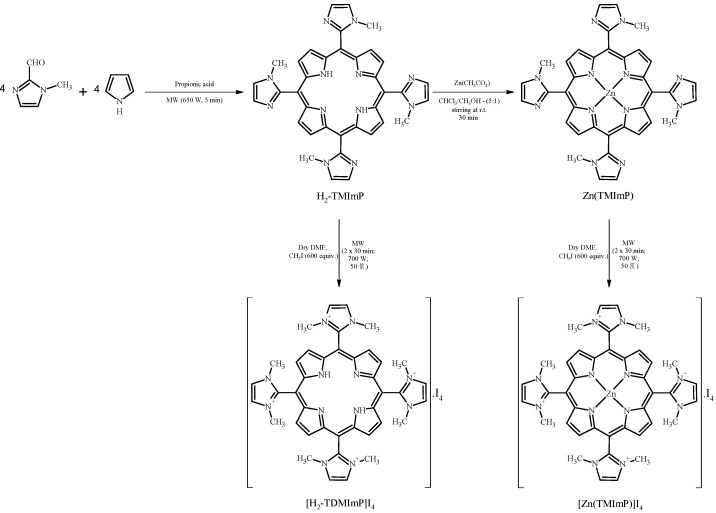
Schematic view of the synthetic route adopted in this work for porphyrin synthesis.

The synthesis of the starting porphyrin and its derivative is simple and straightforward, as outlined in Scheme 1 (see Experimental section), and examples of the potential application as reported in this work, have been put forward for a cationic free-base porphyrin [16] and for its manganese (III) complex [17]. In connection with such applications a US patent assigned to Incara Pharmaceuticals has been submitted [18].

As reported [19], aldehydes containing heteroatoms are not so reactive under Lindsey conditions and, therefore, the Adler conditions are strongly recommended to perform the synthesis. Unfortunately, the unsubstituted imidazolylporphyrin is not easily obtained. However, the corresponding *N*-methyl-substituted derivative is easily obtainable as a mixture of atropoisomers, which might be caused by the presence of methyl groups at pseudo-*ortho*-positions in relation to the porphyrin plane [20,21]. Consequently, the ^1^H-NMR spectrum shows a multifold profile instead of singlet peaks. Our ^1^H-NMR data agree with this finding.

The MALDI TOF/TOF mass spectrometry analysis provided a mass of 631.7 Da (assigned as [M+H]^•+^), whereas the calculated value for C_36_H_30_N_12_ is 630.7 Da, thus confirming the identity and purity of the material. In addition, HRMS analysis yielded a single and intense peak at 631.28060 Da, in complete agreement with the mentioned formula.

**Figure 2:**
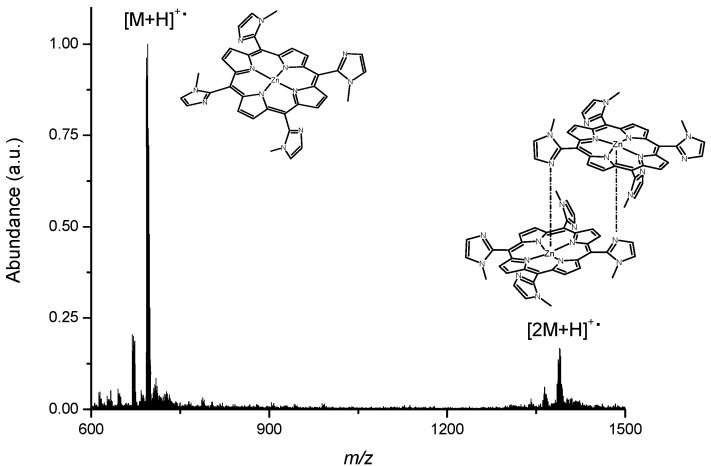
MALDI TOF/TOF spectrum for Zn(TDMImP).

The ^1^H-NMR of Zn(TDMImP) was not so conclusive due to the strong aggregation in solution reported previously. The MALDI TOF/TOF mass spectrometry analysis (Figure 2) shows the [M+H]^•+^ as being 693 Da (for ZnC_36_H_28_N_12_) and a value of 1388 Da, corresponding to dimeric species. The aggregation for this kind of porphyrin seems to be related to the intermolecular self-assembly of a free orbital from zinc of one molecule with a pair of free electron from imidazolyl nitrogen of another molecule [22]. For these cationic compounds, the MALDI TOF/TOF mass spectrometry analysis showed the ion [M-45]^+^ as a predominant peak in both compounds. This ion corresponds to the loss of methyl groups [23].

### 2.2. Spectroscopic Measurements and Quantum Chemical Studies

#### 2.2.1. Absorption spectrum

**Figure 3:**
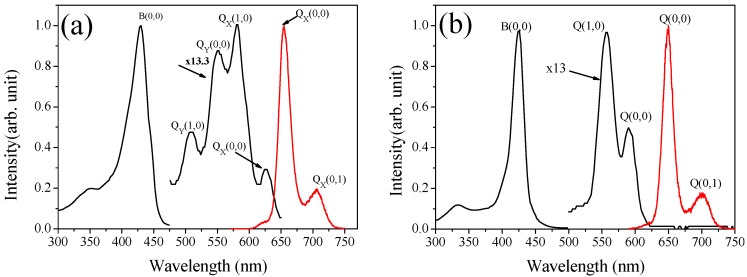
(**a**) Absorption (black line) and fluorescence (red line) spectra of H_2_-TDMImP, at 298 K; (**b**) Absorption (black line) and fluorescence (red line) spectra of Zn(TDMImP), at 298 K.

The absorption spectra, shown in Figure 3, were acquired in anhydrous ethanol solution for the investigated porphyrin derivatives.

Both compounds present a single intense maximum attributed to a B (Soret) band [24], located at 430 nm and 426 nm for H_2_-TDMImP and Zn(TDMImP), respectively, and a less intense blue-shifted absorption band around 350 nm. Like brominated porphyrins [25], our samples also show a great B-Band solvent polarity sensitivity in both compounds. Table 1 and Table 2 summarize the maximum positions of the B-band absorption peaks, as measured for both compounds in different solvents. 

**Table 1:** Absorption peaks for H_2_-TDMImP in different solvents. In parenthesis, the calculated λ_max_ (TD-DFT) in ethanol.

Solvent	λ_max_ (Soret) (nm)	λ_max_ (Q band)(nm)			
		Q_y_(1,0)	Q_y_(0,0)	Q_x_(1,0)	Q_x_(0,0)
Dimethylformamide	435	546	559	585	625
Dimethylsulfoxide	417	511	544	581	633
Acetonitrile	429	507	561	595	632
2-Propanol	431	508	554	582	625
1-Propanol	433	507	554	583	625
Ethanol	429 (**359**)	509	551	581 (**525**)	627 (**560**)
Methanol	426	507	548	581	631
Ethylene glycol	412	508	543	581	634
Water	406	506	540	578	630

**Table 2:** Absorption peaks for Zn(TDMImP) in different solvents. In parenthesis, the calculated λ_max_ (TD-DFT) in ethanol.

Solvent	λ_max_ (Soret) (nm)	λ_max_ (Q band) (nm)	
		Q(1,0)	Q(0,0)
Dimethylformamide	432	561	596
Dimethylsulfoxide	425	555	590
Acetonitrile	430	561	596
2-Propanol	427	560	593
1-Propanol	428	559	593
Ethanol	425 (**376**)	557	591 (**530**)
Methanol	422	555	589
Ethylene glycol	424	554	589
Water	417	551	586

**Figure 4:**
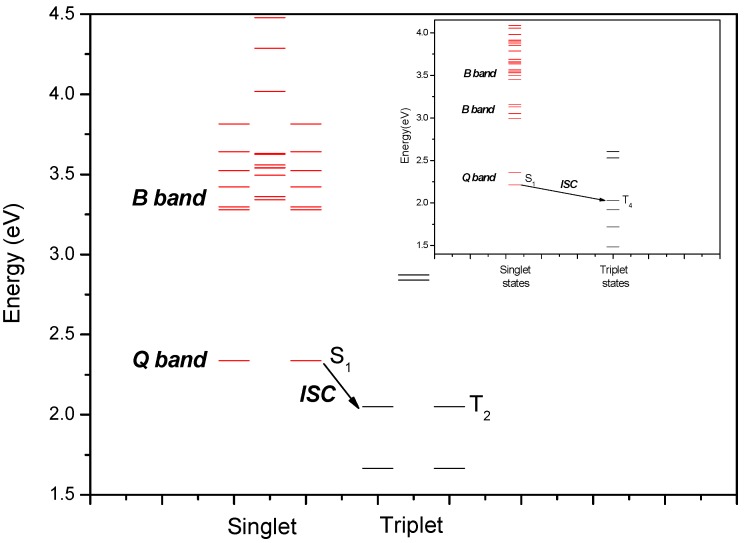
State diagram for Zn(TDMImP). The inset shows the state diagram for H_2_-TDMImP. These diagrams were calculated in ethanol (TD-DFT/IEFPCM) using symmetry constraints. Red lines stands for singlet states, black lines stands for triplet states.

The assignment of the blue shifted band associated with the Soret band has been the source of much controversy [26,27,28]. Yu and coworkers found no significant difference in the emission decay parameters when the porphyrin was excited at the blue shifted and the Soret band [26]. In addition, Liu and coworkers also shown that a significant fraction of the intensity of this absorption band is related to the vibronic structure associated with the Soret absorption [27]. The state diagrams obtained by the TD-DFT calculation (see Figure 4) suggest that both compounds present a considerable complexity for the involved electronic transitions in the B band and its satellite band regions. The occurrence of a large number of very near electronic states between the excitation peaks attributed to both absorption bands result in a large density of electronic states. The combination of these electronic states and the probable association between the vibronic structure of these porphyrins and solvents agree with the proposition that the blue-shifted absorption band is a satellite band related to the vibronic structure of the Soret band [26,27].

In addition, theoretical data also suggest the occurrence of several doubly degenerated singlet and triplet excited states of Zn(TDMImP), a consequence of the coordination of Zn^2+^ at the center of the macrocycle, see Figure 4. Some of the molecular orbitals associated with the B and blue-shifted satellite bands have equivalent symmetry, resulting in a considerable number of possible electronic transitions. These data highlight six transitions with expressive transition probabilities due to the nearly-degenerated character of the excited states (see Table 1 at Supplementary Material).

Similar to what was seen for Zn(TDMImP), the TD-DFT data for the free base indicate that the B band and its blue-shifted satellite band involve a large set of molecular orbitals, some of them of equivalent symmetry, resulting in a considerable number of possible associated electronic transitions. Furthermore, the TD-DFT data also point to six transitions with expressive transition probabilities (see Table 2 at Supplementary Material). On the other hand, for the free base, the occurrence of any degenerate state into the evaluated energy range is not suggested by the TD-DFT/SCRF data, as depicted in the inset of Figure 4. 

Besides the Soret band, a set of Q sub-bands, showing much smaller absorption intensity, is also observed [24,27], see Figure 3. Four Q sub-bands (Q_y_(1,0), Q_y_(0,0), Q_x_(1,0) and Q_x_(0,0)) are assigned to the free base, and a pair of degenerated excited states with x and y polarization (Q(1,0) and Q(0,0)) are related to Zn(TDMImP). For Zn(TDMImP), the occurrence of these degenerate excited states is a consequence of the square symmetry introduced by the metal. 

For Zn(TDMImP) (D_4h_), the Q band, doubly degenerated, involves a pair of π,π* transitions (see Table 3 at the Supplementary Material). In despite of this, the estimated energy gap between the molecular orbitals HOMO (^1^a_1u_) and HOMO-1(^1^a_2u_), 33.39 kJ mol^−1^ (0.3461 eV), calculated from their orbital energies, is sufficiently large so that no degeneracy occurs between these orbitals. These MO interact with two exactly degenerate lowest unoccupied molecular orbitals (LUMO and LUMO+1, both of ^1^e_g_ symmetry). This combination leads to a configuration interaction between the two excited electron configurations at their lowest energy, resulting in two π,π* (^1^a_2u_^1^e_g_ and ^1^a_1u_^1^e_g_) degenerated states. The transitions starting from ground state ((1)^1^A_1g_) to the ^1^E_u_ (x, y polarized) states are allowed by dipole [28]. However, the different orbital symmetries involved in these transitions and the degeneracy of these states imply in the weak absorption observed [29,30]. From these data we also observe that an energy gap of 27.82 kJ mol^−1^ between the first singlet excited state (S_1_) and its adjacent triplet state could be estimated for this compound in ethanol (see Figure 4).

**Figure 5:**
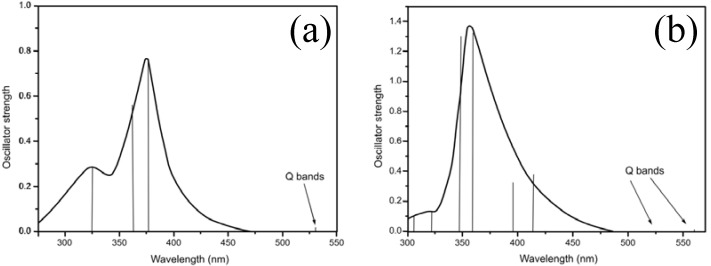
Representation of the TD-DFT UV-Vis excitation spectrum of Zn(TDMImP) (**a**), and H_2_-TDMImP (**b**), calculated under a SCRF procedure (IEFPCM, solvent = ethanol).

Differently from the metallocomplex, the Q band for H_2_-TDMImP (D_2h_) does not present any degenerated states, as can be seen in the inset of Figure 4. This band also involves a pair of π,π* transitions and the energy gap (14.24 kJ mol^−1^) estimated for the first two adjacent singlet states, related to the Q band, which are evidences that they are non degenerated. As occurs for Zn(TDMImP), the different orbital symmetries involved in the transition imply in the weak absorption observed for the Q band (see Table 4 at Supplementary Material). Figure 5 presents the graphical representation, based on the TD-DFT/SCRF data, of the excitation spectrum for both compounds.

They show a good similarity with the experimental absorption spectra. The peak wavelengths corresponding to the Q and B bands are blue-shifted in respect to the experimental data, with values about 16% higher in energy, an acceptable discrepancy which should be attributed to at least three causes: (i) the implicit limitations of the SCRF models in providing a strict description of solute-solvent interactions [31,32]; (ii) the dependence between the used radii to build the cavities that contain the solute and the vertical excitation energies [32]; (iii) the inherent limitations of the selected functional [33,34].

The TD-DFT/SCRF data suggest the existence of very low intensity transitions related to the Q band for both compounds. The data suggest that for Zn(TDMImP), the absorption peak, as discussed previously, is related to Q_x_(0,0) and Q_y_(0,0), which present equivalent oscillator strengths (f_1_ = f_2_ = 0.0208), different from the experimental data for which the Q(0,0)/Q(1,0) intensity ratio is 1:2. For H_2_-TDMImP, the TD-DFT data were unable to show the intensities ratio between the two Q bands. The ratio found between Q_x_(0,0) and Q_x_(1,0) is about 5:1 (f1 = 0.0140 and f2 = 0.0028), when the expected is near 1:3. 

#### 2.2.2. Fluorescence spectrum

As occurs for this class of compounds, the fluorescence spectrum (Figure 3) is related to the two less energetic Q bands, Q(1,0) and Q(0,0), respectively, showing, for Zn(TDMImP) in ethanol, emission maxima at 591 and 643 nm, and for the free base, emission maxima at 604 and 657 nm. The fluorescence spectra were obtained in ethanol at 298 and 77K (not shown here), exciting both compounds in the absorption maximum of their respective B bands. Similar fluorescence spectra can be obtained in other excitation wavelengths of energies lower than the B band, including the Q bands. No fluorescence signal attributed to the B band [26,27] was detected in the experiments.

The fluorescence intensities are usually very low for both compounds, resulting in very small fluorescence quantum yields (Table 3). At 77 K, these compounds present expressive Φ_F_ values and strongest fluorescence signals. The Φ_F_ and τ_F_ estimated in anhydrous ethanol are 0.65 and 15 ns, respectively, for H_2_-TDMImP and 0.27 and 3 ns, for Zn(TDMImP). 

**Table 3:** Fluorescence quantum yields for S_1_→S_0_ transition at 298 K for the studied compounds in different solvents.

Solvent	Φ_F_			E_T_(30)/kcal mol^−1^
	H_2_-TDMImP		Zn(TDMImP)	
N,N–Dimethylformamide	0.004	0.001		43.2
Dimethylsulfoxide	0.008	0.010		45.1
Acetonitrile	0.003	0.002		45.6
2-Propanol	0.006	0.003		48.4
1-Propanol	0.007	0.004		50.7
Ethanol	0.007	0.003		51.9
Methanol	0.008	0.008		55.4
ethylene glycol	0.006	0.011		56.3
Water	0.0004	0.004		63.1

As expected for this class of compounds relatively small Stokes shifts are observed in their fluorescence spectra. For the free base the calculated Stokes shift related to 0→0 transition is 710 cm^−1^. For Zn(TDMImP), the value is more expressive: 1,541 cm^−1^ for 0→0 transition. 

This effect does not present a strict dependence on the polarity of the studied solvents, for both compounds, see Table 3. It is probably correlated to very small structural changes occurred after the molecular relaxation of the excited state into the solvent cage. Since both compounds must be sufficiently solvated in the ground state due to their characteristics, significant changes in the solvation are not expected in the excited state. Thus, an additional relaxation of the first excited state due to the solvation of these molecules is expected to be minimal. In other words, the molecular structure of the relaxed form, in both compounds, in the first excited state, does not differ significantly from the ground state geometry. Consequently, it is expected that the relaxed molecular structure of the first excited state should hold similar characteristics of the Franck-Condon state, and that the ordering of the electronic states in the relaxed S_1_ state suffer only minimal changes.

## 3. Experimental

### 3.1. General

In all experiments, solutions containing the free base 5,10,15,20-tetrakis(1,3-dimethylimidazolium-2-yl) porphyrin tetraiodide salt ([H_2_TMImP]^4+^) or its Zn(II) complex ([Zn(TMImP)]^4+^) were prepared in different solvents, with concentrations ranging from 10^−7^ to 10^−6^ mol dm^−3^, to minimize the possibility of aggregation. 

### 3.2. Synthesis and Characterization

For the synthesis of these two derivatives, all solvents and reagents were used as received, except for pyrrole, which was distilled before use. Propionic acid and DMF were purchased from Merck. Zinc acetate, iodomethane, 1-methylimidazole-2-carboxaldehyde and pyrrole were obtained from Aldrich. 

For microwave assisted organic synthesis (MAOS), a Milestone MycroSinth multimode microwave device equipped with temperature controllers (an infra-red and a bulk temperature sensor) was used rather than a pressure controlling device (when syntheses were performed in a closed vessel). The free base porphyrin, 5,10,15,20-(1-methylimidazol-2-yl)-21H,23H-porphyrin, H_2_TMImP, was obtained as a mixture of atropoisomers from pyrrole and 1-methylimidazole-2-carboxaldehyde in propionic acid under microwave (MW) irradiation, according to the method already described [35,36,37,38,39,40] (see Scheme 1). The purification of the reaction product was done preferably by crystallization, by adding acetone. About three days later, the crystals of a shiny dark purple were separated by filtration.

The metallocomplex was prepared in a simple way. A chloroform solution (5.00 mL) of H_2_-TMImP (50.0 mg; 0.0793 mmol) was prepared. Separately, zinc acetate (45.4 mg; 0.247 mmol) was dissolved in methanol (1.00 mL). Then, the two solutions were mixed at room temperature under stirring. The complexation was monitored by spectrophotometry (UV/Vis) and thin-layer chromatography (TLC). Once completed (30 min), the solvent was removed at reduced pressure and the material was dissolved in chloroform and washed with sodium carbonate solution. The organic layer was dried using anhydrous sodium sulfate and crystallized in CHCl_3_/hexane (1:9). 

The methylation was carried out under MW irradiation for free-base and zinc complex (see Scheme 1). The neutral porphyrin (usually 25.0 mg) was dissolved in dry dimethylformamide (DMF) in a specific microwave reactor equipped with temperature and pressure controlling devices. Then, with a syringe, the iodomethane (CH_3_I) was added (usually 3.00 mL). Irradiation was left for two pulses of 30 minutes using 700 W as initial power. The temperature was controlled up to 50 °C by a computer program. 

After completion of the reaction, diethyl ether was added to the reaction flask to precipitate the porphyrin derivative. Recrystallization was carried out in methanol/dichloromethane for the free base 5,10,15,20-tetrakis-(1,3-dimethylimidazolium-2-yl) porphyrin tetraiodide [H_2_TDMImP]I_4_, and in methanol/acetone for 5,10,15,20-tetrakis-(1,3-dimethylimidazolium-2-yl) porphyrinate zinc (II) tetraiodide, [Zn(TDMImP)]I_4_ (Scheme 1). The yields related to these cationic compounds were 88% and 80%, for free base and Zn(II) complex, respectively. It is worth mentioning that, under MW irradiation, the yield is similar when the synthesis is performed under conventional heating. However, the time required to prepare the porphyrin is only five minutes [35] whereas four hours are recommended when using oil bath as the heating source [16].

The ^1^H-NMR spectra were recorded in a Bruker Avance 300 instrument at 300.13 MHz. TMS was used as the internal reference. The chemical shifts are expressed in δ (ppm). The ^1^H-NMR spectral analysis (DMSO-d*_6_*) gave the following characteristic signals: -3.25 (s, porphyrin N-H, 2H), 3.75 (s, imidazolium 1,3-N-CH_3_, 24H), 8.55 (s, imidazolium H*^4^* and H*^5^*, 8H) and 9.42 (s, pyrrole *β*-H, 8H), for [H_2_TDMImP]I_4_, and 3.71 (s, imidazolium 1,3-N-CH_3_, 24H), 8.53 (s, imidazolium H*^4^* and H*^5^*, 8H) and 9.24 (s, pyrrole *β*-H, 8H), for [Zn(TDMImP)]I_4_. The mass spectrometry analysis was carried out in a 4800 Maldi TOF/TOF Analyzer, Applied Biosystems.

### 3.3. Spectroscopic Measurements

The absorption and emission spectra were recorded in different solvents (DMF, DMSO, acetonitrile, 2-propanol, 1-propanol, ethanol, methanol, ethylene glycol and water), analytically pure or spectroscopic grade, used without further drying or purification, whereas fluorescence measurements were done using anhydrous ethanol exclusively.

The UV/VIS absorption and emission spectra were respectively recorded using a Shimadzu UV-Vis1650 PC spectrophotometer and a Hitachi F-4500 spectrofluorimeter, equipped with accessories for measurements under low temperature, being the fluorescence spectra obtained using a right angle configuration. The maximum absorption wavelength of the Soret band was used to excite samples. The absorbance at this wavelength was maintained below 0.100 to avoid light reabsorption effects. 

The fluorescence quantum yields were estimated from corrected fluorescence spectra, using the secondary standard method [41]. Rhodamine B in methanol (Φ_F_ = 0.52 at 298 K; λ_exc _= 532 nm; λ_em _= 565.8 nm) was used as fluorescence standard in all measurements [42]. Low-temperature luminescence measurements were done at 77 K, using liquid nitrogen, in solutions prepared in anhydrous ethanol previously deaerated by means of an argon flux. 

The fluorescence decay measurements were performed as previously described in reference [43], through the use of an apparatus based on the time correlated single photon counting method. The excitation source was a titanium–sapphire laser, whose frequency was doubled to 465 nm in a LBO crystal, pumped by the second harmonic of a diode-pumped Nd:YVO4 laser. The signal was detected next to the fluorescence maximum, at 640 nm.

### 3.4. Quantum Chemical Calculations

Both compounds had the ground state geometry of the cationic part optimized, using the B3LYP functional from Density Functional Theory (DFT) [44]. These calculations were performed under a self-consistent reaction field approach (SCRF), simulating the solvation of the compounds in ethanol and DMSO, using the IEFPCM model [31,32]. The structures were optimized using D_2h_ and D_4h_ symmetries, respectively, for H_2_TMImP and Zn(TDMImP). The 6-31G(d,p) atomic basis set was used to define carbon, hydrogen and nitrogen, while LANL2DZ pseudo-potential was used for zinc. These calculations were followed by analytical vibrational frequency computations, in order to verify the nature of the optimized state. The optimizations were restricted only to the cationic portion of these compounds since they tend to become iodized under the level of dilution employed.

With the optimized structures, the excitation energies for the first twenty-five excited singlet and six triplet states, and their respective oscillator strengths in ethanol, were calculated using the Time-Dependent Density Functional Theory (TD-DFT), the same hybrid functional, and the DGDZVP2 valence double-ζ atomic basis-set, combined with the IEFPCM SCRF approach.

## 4. Conclusions

The synthesis of the porphyrins presented in this study is simple and straightforward, yielding a reasonable amount of the desired materials. Different from conventional porphyrin derivatives, these compounds are based on five-membered rings at the *meso*-position. Such feature gives rise to different optical and electronic properties for these materials.

Both compounds presented the typical Soret and Q absorption bands observed for porphyrins, with a Soret band sensitive to solvent polarity. Moreover, a satellite band blue-shifted in relation to Soret band is observed for both compounds. The combination of the electronic states of these two bands and the probable association between the vibronic structure of these porphyrins and solvents agree with the proposition that the blue-shifted absorption band is a satellite band related to the vibronic structure of the Soret band. The calculations also point out to the existence of a great amount of degenerated triplet and singlet exited states for zinc species.

Typically, the Φ_F_ is very low for both compounds at 298 K. At 77 K, as expected an increase in the Φ_F_, for instance, in ethanol we measured 0.65 for free base and 0.27 for zinc porphyrin. The fluorescence decay time measured at room temperature is 15 ns for H_2_TMImP and 3 ns for Zn(TDMImP).
